# New insights into the tonoplast architecture of plant vacuoles and vacuolar dynamics during osmotic stress

**DOI:** 10.1186/1471-2229-5-13

**Published:** 2005-08-04

**Authors:** Daniel Reisen, Francis Marty, Nathalie Leborgne-Castel

**Affiliations:** 1 UMR PME INRA/CNRS/Université de Bourgogne BP 47870, boulevard Gabriel, 21078 Dijon Cedex, France; 2 Department of Molecular Biology and Genetics, 321 Biotechnology Building, Cornell University, Ithaca, NY 14853, USA

## Abstract

**Background:**

The vegetative plant vacuole occupies >90% of the volume in mature plant cells. Vacuoles play fundamental roles in adjusting cellular homeostasis and allowing cell growth. The composition of the vacuole and the regulation of its volume depend on the coordinated activities of the transporters and channels localized in the membrane (named tonoplast) surrounding the vacuole. While the tonoplast protein complexes are well studied, the tonoplast itself is less well described. To extend our knowledge of how the vacuole folds inside the plant cell, we present three-dimensional reconstructions of vacuoles from tobacco suspension cells expressing the tonoplast aquaporin fusion gene *BobTIP26-1::gfp*.

**Results:**

3-D reconstruction of the cell vacuole made possible an accurate analysis of large spanning folds of the vacuolar membrane under both normal and stressed conditions, and suggested interactions between surrounding plastids. Dynamic, high resolution 3-D pictures of the vacuole in tobacco suspension cells monitored under different growth conditions provide additional details about vacuolar architecture. The GFP-decorated vacuole is a single continuous compartment transected by tubular-like transvacuolar strands and large membrane surfaces. Cell culture under osmotic stress led to a complex vacuolar network with an increased tonoplast surface area. In-depth 3-D realistic inspections showed that the unity of the vacuole is maintained during acclimation to osmotic stress. Vacuolar unity exhibited during stress adaptation, coupled with the intimate associations of vacuoles with other organelles, suggests a physiological role for the vacuole in metabolism, and communication between the vacuole and organelles, respectively, in plant cells. Desiccation stress ensuing from PEG treatment generates "double" membrane structures closely linked to the tonoplast within the vacuole. These membrane structures may serve as membrane reservoirs for membrane reversion when cells are reintroduced to normal growth conditions.

**Conclusion:**

3-D processing of a GFP-labeled tonoplast provides compelling visual constructions of the plant cell vacuole and elaborates on the nature of tonoplast folding and architecture. Furthermore, these methods allow real-time determination of membrane rearrangements during stresses.

## Background

Space-filling, turgor-driving vacuoles must have originated at a very early stage of biological evolution and have subsequently evolved to undertake various functions, well-known in algae, fungi (including yeast), and plants [1]. Many of the advanced, complex functions operate on or are closely associated with the vacuolar sap-bounding membrane, i.e. the tonoplast. A detailed structural study of dynamic events mediated by the tonoplast should extend our knowledge of its cellular functions. In the past, *in vivo *observations of the vacuolar membrane were restricted by the resolution of light microscopy. Like other cell components of a size below the limit of resolution of the light microscope, the tonoplast has been studied mostly by electron microscopy. However, this permits only post-mortem observations of thin sections from rapidly frozen freeze-substituted or chemically fixed cells [2], or replicas of fracture faces from fast-frozen cells [3,4]. Although these studies have provided invaluable insights into the organization and biogenesis of the tonoplast, analysis of thin specimens provides only limited information about its spatial architecture. High voltage electron microscopes have the ability to retrieve large amounts of information from thick sections through fixed cells, but depend on the use of a restricted number of selective (non-vital) "staining" techniques to overcome the decrease in image contrast typically seen at high accelerating voltages [4-6]. As such, the use of these different techniques makes it difficult to study and understand the dynamic changes of the vacuolar membrane in living cells.

The confocal fluorescence microscope can eliminate out-of-focus blur. Three-dimensional (3-D) data from intact biological specimens can therefore be obtained by non-invasive optical sectioning [7]. A new revolution in microscopy came over a decade ago with the use of "green fluorescent protein" (GFP) from jellyfish *in vivo *[8]. GFP and its variants are now frequently used to generate a fluorescent organelle, e.g. mitochondria [9], chloroplasts [10] or components of the secretory system [11], allowing one to study their dynamics in living cells. Furthermore, several studies present data of GFP-labeled tonoplasts [12-21], of which only a few followed tonoplast dynamics [20,21].

Since the best resolution of living cells is obtained with laser scanning confocal microscopy images, plant-compatible GFP cDNA [22] was fused, in frame, to the 3' end of the cauliflower BobTIP26-1 tonoplast-specific aquaporin cDNA cloned in our laboratory [23]. The resultant chimeric gene was expressed in tobacco cells (var. Wisconsin 38) grown in suspension, which display a GFP-decorated tonoplast [17]. This method of in vivo labeling was used to 3-dimensionally reconstruct the tonoplast of cells at different stages of growth and under various osmotic stresses. Our data show that the GFP-decorated vacuole in tobacco suspension cells is a single continuous compartment. Furthermore, osmotic stress conditions yield a greater tonoplast surface area while maintaining vacuole unity. Moreover, PEG treatment generates spherical structures that are associated with the inner side of the vacuolar membrane. We propose that these structures serve as membrane reservoirs necessary for membrane reorganization after cells are returned to normal growth conditions.

## Results

### Three-dimensional reconstruction of the vacuole

We have recently expressed a tonoplast aquaporin fusion gene *BobTIP26-1::gfp *under the CaMV35S promoter in tobacco cells [17]. The proper targeting of the resultant fusion protein to the tonoplast was confirmed by laser scanning confocal microscopy (Additional file 1). Tobacco cells expressing the chimeric protein were used for 3-D reconstructions of the cell vacuoles.

**Figure 1:**
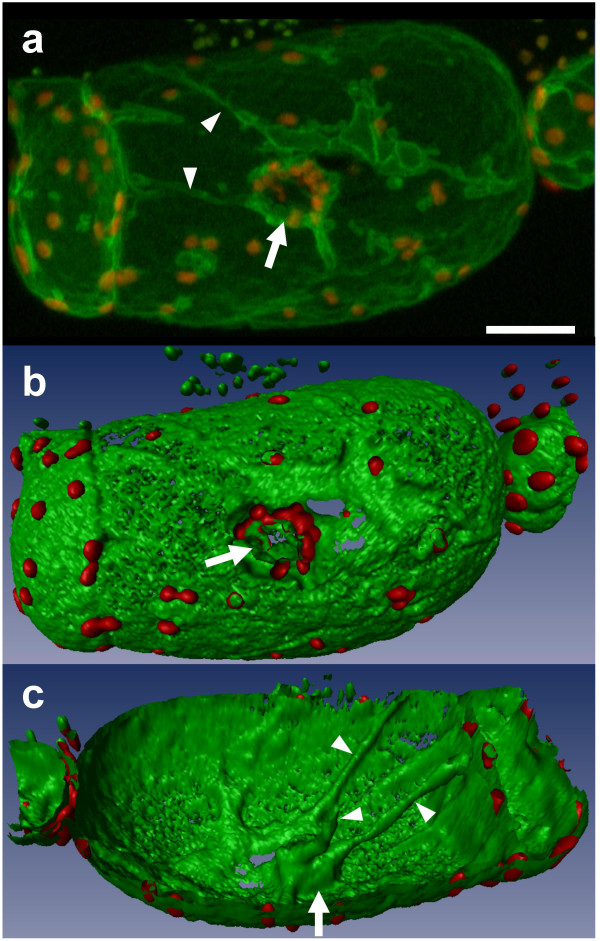
**Three-dimensional vacuole reconstruction of a vacuolated *BobTIP26-1::gfp *expressing cell 7 days after subculture. **(a) Projection view of 40 confocal serial pictures corresponding to the half depth of the cell (i.e. 40 μm). Bar = 25 μm. (b) 3-D view after isosurface extraction showing the protoplasmic side of the vacuole. (c) Interior view of the vacuole after isosurface extraction. Green and red correspond to the tonoplast and the chloroplasts, respectively. Arrow: nuclear pouch; arrowheads: transvacuolar strands. The missing domains of the tonoplast surface in (b) and (c) result from an under-sampling of confocal images. The rendering of a completely smooth 3-D view would have required use of additional intermediate sections.

**Figure 2:**
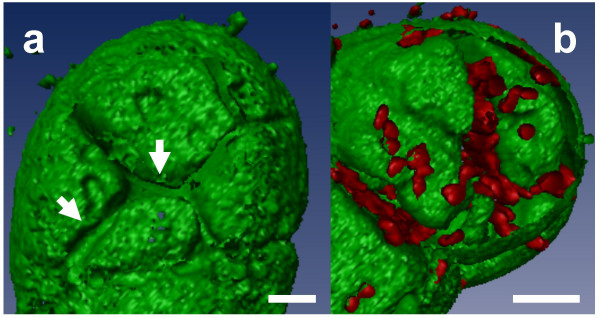
**Outside shape of a vacuole. **(a) Furrows on the outer part of the tonoplast (arrows) revealed by 3-D surface rendering. (b) Chloroplasts – in red – fill up the furrows on the tonoplast surface. Isosurfaces of both channels are displayed. Bar = 10 μm.

In 7 day-old turgid cells, vacuoles occupy almost the entire cell volume. In a projection view, where 40 optical sections of such cells were merged (z-step = 1 μm; Fig. 1a), a 3-D relief of the vacuole was barely observable, and structural details of the tonoplast were not easily discerned. Realistic 3-D pictures of the tonoplast (Fig. 1b, 1c) were obtained after isosurface extraction, a procedure in which volume images were converted into geometric surfaces [24] by using the 3-D visualization software Imaris 2.7 (Bitplane AG, Switzerland). The intravacuolar surface of the tonoplast was readily scrutinized when only "half vacuoles" were reconstructed. As expected, red autofluorescent chloroplasts were seen at the outer surface of the GFP-labeled membranes, confirming their cytoplasmic localization (Fig. 1b). Chloroplasts were also clustered around the nucleus, where the tonoplast forms a cavity (Fig. 1, arrow). Labeled transvacuolar cytoplasmic strands were seen radiating throughout the vacuole, extending from the nuclear region to the cell periphery (Fig. 1c, arrowheads). Furrows were also occasionally observed on the cytoplasmic side of the tonoplast (Fig. 2a, arrows). When the isosurface mode was used to visualize simultaneously chloroplasts and tonoplasts within large vacuolated cells, numerous chloroplasts were seen lying within these furrows (Fig. 2b).

**Figure 3:**
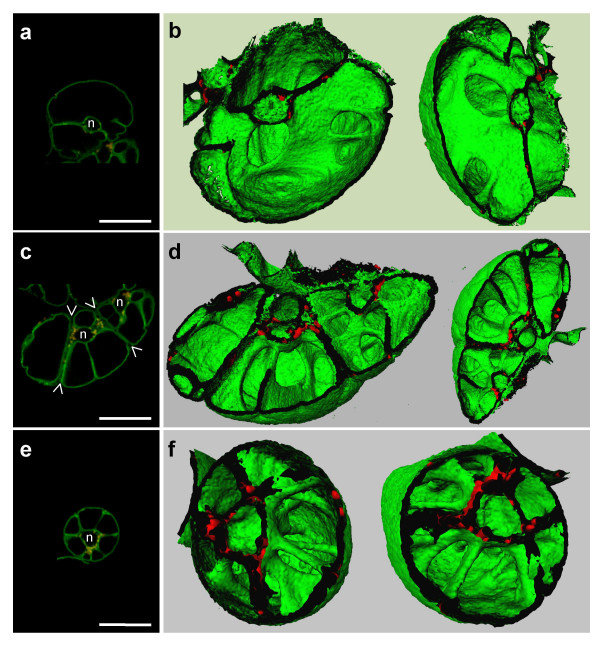
**Vacuole continuity through gaps inside tonoplast sheets. **(a, c, e) Single confocal images. The focal plane chosen corresponds to the one through the vacuoles' center. Arrowheads (>) in (c) delimit joined cells. n, nuclear region. Bars = 50 μm. (b, d, f) 3-D representation of the vacuole halves after isosurface extraction. Notice the openings in the vacuolar layers (b and d) and the many transvacuolar strands emanating from the nuclear region (f).

We observed cells of different sizes and shapes in the non-synchronized tobacco cell suspension. While confocal images suggest the existence of several vacuoles inside each cell (Fig. 3a, 3c, 3e), 3-D representations of each cells' tonoplast, and the visualization of openings within the vacuolar lumens clearly support a "one cell, one vacuole" model, i.e. vacuolar continuity exists between GFP-labeled vacuolar compartments within a cell (Fig. 3b, 3d, 3f). Indeed, large surface areas of tonoplast may transect the vacuole, but gaps exist, allowing continuity of the vacuolar interior. The three joined cells (Fig. 3c) each possesses such transvacuolar layers, but 3-D representations show that they do not define discrete vacuoles in each cell (Fig. 3d). An animated sequence of these 3-D reconstructed vacuoles is shown in the Additional File 2. What seemed to be individual vacuolar cavities in the cells observed by confocal microscopy was seen as a single vacuolar compartment by 3-D reconstruction. Careful analysis through a tomogram of a protoplast prepared from *BobTIP26-1::gfp *expressing tobacco cells is also in accord with this feature (Additional File 3).

Cellular architecture details that are evident as a result of 3-D reconstructions include the demonstration of a vacuole with numerous transvacuolar strands, outwardly radiating from the nuclear region to the cell periphery (Fig. 3e, 3f). 3-D reconstructions also showed that chloroplasts do not merely surround the nucleus; their distribution also parallels that of the transvacuolar strands.

### Tonoplast behavior during plasmolysis

**Figure 4:**
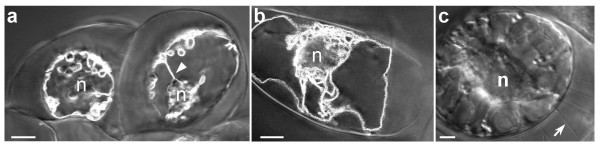
**Cell perfusion with MS supplemented with 0.6 M mannitol. **(a) A confocal fluorescent image merged with a Nomarski interference contrast image of convex plasmolysed cells. Arrowhead: transvacuolar strand. Bar = 10 μm. (b) A confocal fluorescent image merged with a Nomarski interference contrast image of a concave plasmolysed cell. n, nucleus. Bar = 10 μm. (c) A Nomarski interference contrast image of a plasmolysed cell. Hechtian strands (arrow) attach the protoplast tightly to the cell wall. n, nucleus. Bar = 5 μm.

We further analyzed the behavior of the labeled tonoplast during plasmolysis. Tobacco suspension cells were bathed in culture media supplemented with different osmotica (170 mM sodium chloride, 0.6 M mannitol or 0.5 M sorbitol) to induce plasmolysis-deplasmolysis cycles. Plasmolysis occurred 30 to 60 sec after the osmoticum entered the perfusion chamber. The phenomenon preferentially started at the cell corner, with complete plasmolysis occurring after 3 to 5 min. Two forms of plasmolysis were observed in tobacco cells, as defined by Oparka [25]. The convex form of plasmolysis was most frequently observed, which results from an even separation of the protoplast from the walls, thereby forming a symmetrical and roughly spherical protoplast (Fig. 4a). Occasionally, the concave form of plasmolysis was observed (Fig. 4b). In this form, concave pockets are formed as the plasma membrane separates from the wall. During both form of plasmolysis, the protoplast was tightly connected to the cell wall by Hechtian strands (Fig. 4c, arrow).

During the plasmolysis step, a peculiar folding of the GFP-labeled tonoplast characterized by complex curling surrounding the nucleus developed within the vacuole (Fig. 4a, 4b). Closer examination of the curled structures revealed that they were slightly more fluorescent than the tonoplast to which they were tethered, an observation that might be due to the joining of two adjacent labeled membranes. Furthermore, the vacuole and the tonoplast remained intact and no vesicle formation was detected. A cellular tomogram through a plasmolysed cell clearly demonstrates the uninterrupted integrity of the tonoplast, its folds and curves being readily visible (Additional File 4). The absence of tonoplast inside the Hechtian strands was confirmed by the absence of labeling (Additional File 4). However, transvacuolar cytoplasmic strands could still be observed inside the vacuoles of plasmolysed (Fig. 4a, arrowhead; Additional File 5) and deplasmolysed cells (Additional file 5). These intravacuolar structures appear to be less flexible than the peripheral tonoplast. Cells were kept plasmolysed for 10 to 15 min before they were bathed again in normal MS culture medium. Plasmolysed cells returned to their normal shape after about 10 to 20 min (Additional File 5), and the curled structures unwound. The position of the nucleus within the cell was nearly constant throughout the plasmolysis-deplasmolysis process.

### Effects of osmotic stress on tonoplast architecture

**Figure 5:**
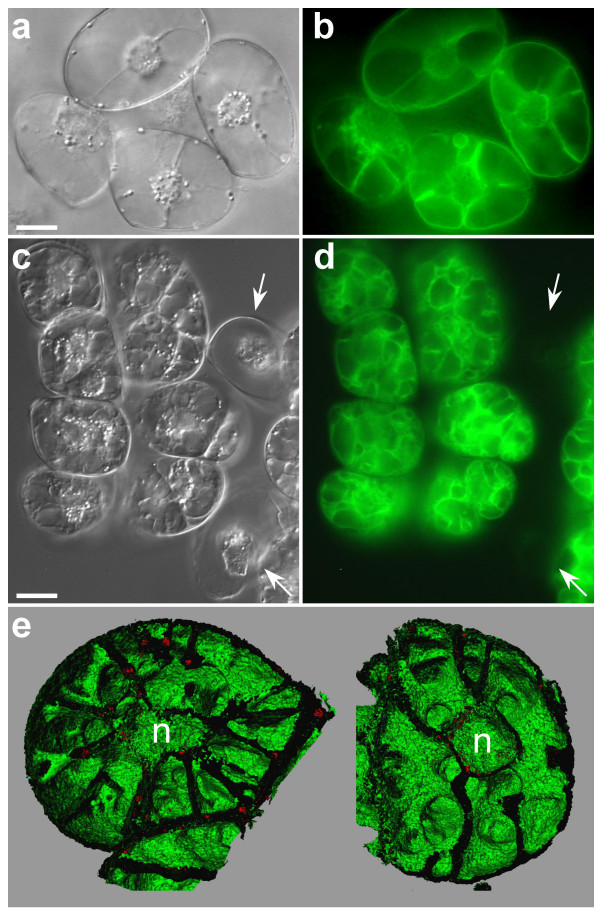
**Normal cells versus osmotic stressed cells. **(a, b) Cells under normal growth conditions. Bar = 25 μm. (c, d) Cells grown in MS supplemented with 0.6 M mannitol (i.e. acclimated cells). Arrows correspond to dead cells (i.e. non-acclimated cells). Bar = 25 μm. (e) 3-D reconstruction of the vacuole after isosurface extraction from acclimated cells cultured in MS supplemented with 0.5 M sorbitol.

Consistent with the results obtained with 3-D reconstructions, we tried to ascertain the tonoplast architecture during osmotic stress culture. Indeed, during NaCl culture conditions of tobacco suspension cells, a vacuolization phenomenon, characterized by a fragmentation of the central vacuole into multiple smaller ones, has been described [26]. To prove the existence of this fragmentation in tobacco cells exhibiting a fluorescent tonoplast, *BobTIP26-1::gfp *expressing cells were subcultured in hyper-osmotic MS medium containing 170 mM NaCl, 0.6 M mannitol or 0.5 M sorbitol, with final water potentials of -1.4 MPa; -1.6 MPa and -1.2 MPa, respectively. Compared with cells subcultured in normal MS medium (Fig. 5a, 5b), cells acclimated in hyperosmotic medium were smaller (Fig. 5c, 5d). [Hereafter, cells which have been grown in media containing osmoticum are referred to as "acclimated cells"]. The extrapolated volumes of cells grown in normal MS and in hyperosmotic medium are on average 175·10^3 ^μm^3 ^and 53·10^3 ^μm^3^, respectively. After acclimation, cell volume decreases ~3.3 times. If the vacuole represents 90% of the cell volume in cells grown in normal MS medium, the original vacuole volume would be 157·10^3 ^μm^3^.

The vacuolization phenomenon could be monitored under Nomarski contrast interference optics (Fig. 5c). Furthermore, this phenomenon was more easily observed with fluorescent labeling of the tonoplast (Fig. 5d). In these cultures, only ~25% of the cells were completely plasmolysed (Fig. 5c arrows), and did not show any fluorescence, indicating that they are non-acclimated, dead cells (Fig. 5d arrows). Additionally, vital staining with neutral red showed that only acclimated cells contained the dye in their vacuoles (data not shown).

To determine if the apparently numerous, independent, small vacuoles of acclimated cells are part of a continuous vacuolar compartment, 3-D reconstructions of the entire vacuole were achieved. The images indicated the existence of a complex vacuolar network. A depth inspection with stereo-viewers showed interconnections between the vacuolar cavities in completely reconstructed vacuoles. Halves of such reconstructed vacuoles are presented in Figure 5e. The nuclear pouch can be seen at the cell center, surrounded by small, interconnected vacuolar cavities. Despite strong evidence for the existence of a solitary vacuolar space, we cannot rule out the possibility that acclimated cells contain small, discrete cavities. However, no small structures of this nature could be discerned using the complicated reconstructions. Since vacuolar cavities are interconnected, we suggest that the tonoplast surface area increases significantly while the continuity of the vacuolar lumen remains unaltered. The software (Imaris 2.7) used for the 3-D reconstructions did not allow quantification of the tonoplast surface area, nor comparison of the tonoplast surfaces of acclimated cells to those of normal cultured cells.

### Tonoplast architecture modifications during dehydrative stress

**Figure 6:**
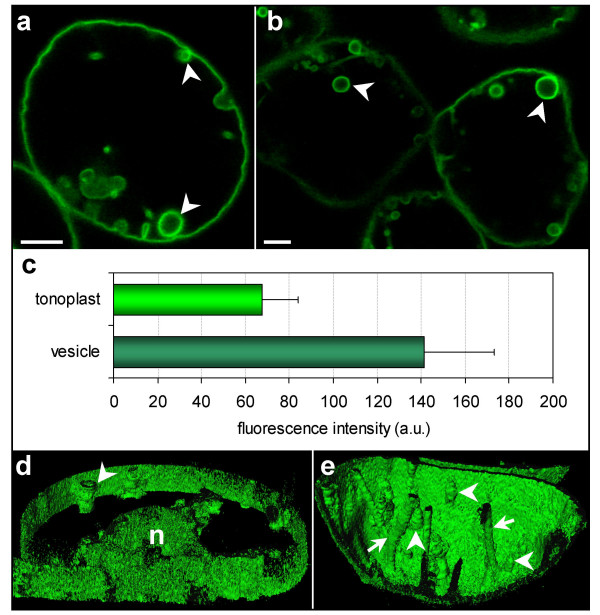
**PEG-stressed cells. **(a, b) Localization of BobTIP26-1::GFP in the membrane of spherical structures (arrowheads) within vacuoles. Bar = 10 μm. (c) A histogram of fluorescent intensity values collected from both the tonoplast of the cell periphery and vesicles. (d) A 3-D slice reconstruction through the vacuole at the position of the nucleus (n). Arrowhead = spherical structure. (e) 3-D reconstruction through a part of a vacuole. Transvacuolar strands are still present in these cells (arrow); spherical structures are primarily fixed onto the tonoplast (arrowheads).

Dehydrative stress was mimicked using the macromolecule polyethylene glycol (PEG_8000_), which passes with either difficulty or not at all through the cell wall [27], thereby reducing the extracellular free water concentration [28]. While an overall increase in membrane surface area occurred in hyperosmotically stressed suspension cells, cells cultured in MS medium, supplemented with 10% PEG_8000_, displayed numerous spherical fluorescent structures of 5–10 μm in diameter, but neither complex membrane rearrangement nor folding. These inner vacuolar structures exhibited a brighter membrane fluorescence compared to that of the peripheral tonoplast (Fig. 6a, 6b, arrowheads). Indeed, the fluorescence intensity of the membrane surrounding these structures was at least twice that of the peripheral tonoplast (Fig. 6c). These spherical structures were also dynamic, moving inside the vacuolar lumen, but seemingly attached to the peripheral vacuolar membrane (Fig. 6d, arrowhead). They were easily distinguishable from the transvacuolar strands, which maintained their morphology (Fig. 6e, arrow). Indeed, the transvacuolar strands transect the lumen of the vacuole from one part of the vacuole to the other, while the spherical structures are tethered to the vacuole perimeter.

## Discussion

### 3-D reconstruction of the vacuole under native conditions

The plant vacuole is a multi-functional organelle [1] which serves as a true *milieu intérieur *[29], playing key roles during cell growth [30], and possibly in the osmoregulation of water during osmotic stress of the cytoplasm [31]. For the purpose of redefining the vacuole architecture, we present the use of 3-D reconstructions of the vacuolar apparatus from cells containing a GFP-labeled tonoplast under "native" conditions. The tonoplast marker system [17] was developed to gain new insights of the tobacco cell vacuole morphology during both normal and osmotic stress growth conditions. A 3-D representation of the vacuole using confocal serial pictures offers the most accurate illustration of how a vacuole is folded within a plant cell. Indeed, previous examination of mitotic BY-2 cells expressing a GFP-labeled syntaxin yielded comprehensive images of vacuolar architecture by reconstructing 3-D surfaces obtained from sequential confocal sections [32]. A side effect of the morphology of the tonoplast due to the over-expression of a membrane protein such as BobTIP26-1::GFP can, a priori, not be excluded, but results obtained for both 35S-GFP-AtVam3p and the lipophilic probe FM4-64 in BY-2 cells revealed that vacuolar morphology was not artificially affected in transgenic BY-2 cells [32]. The fluorescent tonoplast in *BobTIP26-1::gfp *expressing cells appears to be similar to the FM4-64 stained one in BY-2 cells, assuming that vacuolar morphology in *BobTIP26-1::gfp *expressing cells is not altered.

The software used for the representation of the laser confocal microscope data offered two forms of volume visualization: isosurface rendering and direct volume rendering [33]. We chose the isosurface rendering method, in that it allowed us to represent a shaded surface as an easily interpretable 3-D object. However, the two techniques are complementary and the conclusions offered by the final 3-D pictures are equivalent.

A 3-D vacuole structure of BY-2 tobacco cells expressing *TIP::gfp *was first established by Mitsuhashi and co-workers [13], but their representation was mainly a projection view of sequential confocal images that revealed the presence of several large vacuoles folded within the cell. However, as demonstrated in the present study, a projection view may not be as informative as 3-D reconstruction (Fig. 1, Fig. 3). Whereas projection views showed multiple vacuoles folded within a single cell, 3-D reconstructions accurately portray a single vacuolar continuum within cells cultivated under normal growth conditions (Fig. 3). Such continuity of the vacuolar lumen has been described by Palevitz and co-workers [34], who analyzed the vacuole during cell differentiation of *Allium *stomata cells. Although thin sections of these fixed cells observed by electron microscopy revealed individual small vacuoles, a 3-D reconstruction using 0.25 to 0.50 μm thick serial sections, viewed at 100 kV, clearly showed the sections originated from a continuous reticulate network.

The concept of a continuous vacuole is important for the plant cell because the content of the vacuolar sap can thereby flow between all regions of the cell when homeostatic measures are undertaken by stressed cells.

To obtain enhanced resolution of the vacuole, reconstructions based on electron tomograms should be used as exemplified in the data obtained for both *Arabidopsis thaliana *mitotic cell plate formation [35-37] and the Golgi apparatus of animal [38] and yeast [39] cells.

### Tonoplast surface architecture

With 3-D reconstructions of the vacuole, we were able to analyze its surface architecture from a unique perspective. Earlier 3-D renderings of the vacuole-cytoplasm interface of tobacco cell vacuoles showed ripples on the acridine orange labeled vacuole surface [40], which differ from surface furrows we have described. Indeed the vacuole ripples dip into the cytoplasm, but not into the vacuole [40]. We observed the furrows just after isosurface reconstruction and they seem to result from the pressure exerted by some organelles onto the turgid vacuole. The organelles were thus squeezed between the plasma membrane and the tonoplast creating hence the furrows on the cytoplasmic interface with the vacuole. We show here that the 3-D rendering is also useful to visualize interactions between organelles.

### The tonoplast surface is enlarged during osmotic stress

Although membranes are targets of stress-induced cellular damage, and vacuoles are thought to have a central role during stress, few studies of the tonoplast during stress conditions have been reported. We reasoned that scrutinizing the pattern of the tonoplast during different osmotic stresses would build upon our current knowledge of how the cell organizes this membrane during environmental changes. Glycophytic cells, such as tobacco cells (*Nicotiana tabacum *var. Wisconsin 38), were previously analyzed for their ability to acclimate to NaCl [41] and determined to have an increased vacuolization as well as an extensive network of transvacuolar membrane strands [26]. Transvacuolar strands are easily visualized with Normarski optics under a light microscope; however, their composition is more difficult to deduce. Our use of the tonoplast intrinsic GFP-tagged protein that we developed [17] offers *in situ *evidence that transvacuolar strands transecting the vacuole lumen also contain tonoplast associated molecules (Fig. 1, Fig. 3f).

Tobacco cells growing in the presence of diverse osmotica developed an extensive membrane network that appears to transect the vacuole, thereby creating multiple compartments. Observations of similar phenomena were previously described for cells grown during NaCl acclimation [26]. With a 3-D representation, we found that growth in media supplemented with salt, mannitol or sorbitol resulted in transvacuolar strands completely changing their organizational pattern, shifting from a thin shape during normal conditions to larger surface areas. Concomitant with strand pattern alterations was the appearance of several small cavities. These phenomena were observed regardless of the osmoticum used for acclimation. The 3-D picture obtained from acclimated cells clearly demonstrates that the number of vacuoles remains constant while the total tonoplast surface area increases, thereby creating a more complex vacuolar pattern (Fig. 5e). Thus, the ratio of tonoplast surface area to volume of cytoplasm is optimized for exchanges (transport of water and ions) between the cytosol and the vacuole. As the total surface area of tonoplast membrane increases, it follows that the total mass of membrane compounds also increases. It is notable that similar vacuolization changes were observed to occur after hyper-osmotic treatment of wild type tobacco cells; therefore, the increased membrane surface is linked neither to the presence of the aquaporin nor the greater cell size of *BobTIP26-1::gfp *expressing cells [17]. Measurements of the surface areas of the tonoplasts in acclimated cells are complicated by the absence of vacuole shape uniformity. In contrast, the prolate spheroid shape of *Vicia faba *guard cells allows for the determination of both their surface areas and volumes after 3-D reconstruction efforts [42]. In our studies such standardization of conditions was not possible.

### Folding of the plasmolytic vacuole

Notwithstanding the massive changes in vacuolar surface area which accompany plasmolysis, only a few observations on the fate of the vacuole during osmotic contraction have been described in the relevant literature. Plasmolysis-deplasmolysis cycles were realized in a perfusion chamber, allowing observation of fluorescent tonoplast labeled with GFP. During plasmolysis, it was observed that the tonoplast undergoes folding and that the vacuole does not vesiculate into discrete multilamellar vesicles severed from the tonoplast as "sac-like, rod-like or doughnut-shaped structures" as previously described [43]. Furthermore, complete tomography of a plasmolysed cell (Additional File 4) demonstrated that the tonoplast folds in a peculiar way inside the protoplast, rather than being broken into small vesicles, as reported earlier [25]. Such a process seems better suited than vesicle formation for a faster reestablishment of the vacuole during deplasmolysis. No membrane fusion is necessary.

Transvacuolar strands, thin tubular structures that traverse the vacuole, were observed not to break down both during plasmolysis and after deplasmolysis (Additional File 5). Earlier studies showed that transvacuolar strands were stabilized by actin filaments [44,45] and rearranged by myosin motors through their interactions with actin filaments [46]. Despite these observation of strand stability, they have also been described as dynamic and delicate [26]. Our data support the notion that transvacuolar strands exhibit both "strength" and stability, in that their positions remaining static inside the vacuole during abrupt environmental changes (Additional File 5).

### PEG treatment results in spherical structures composed of tonoplast

Surprisingly, spherical structures with twice the fluorescent intensity as the tonoplast were observed inside the vacuole lumen of tobacco suspension cells cultured in MS medium supplemented with 10% PEG_8000_. These structures were similar to those frequently observed in either tonoplast GFP-labeled tobacco leaves [47,48] or in *Arabidopsis *cotyledons [49]. Additionally, such structures have been described in cotyledons, hypocotyls and roots of the *Arabidopsis *vacuolar biogenesis *bub *(bubble-bath) mutants [50], as well as in transiently transformed *Nicotiana benthamiana *plants expressing a GFP fusion protein homologous to a high-affinity tonoplast phosphate transporter [51]. The structures we observed during PEG treatment were independent of the water regulation or over-expression of an aquaporin gene because we, as well as other authors, found similar bulbs in *Arabidopsis *transformed cells with native TIP1;1 promoter (Bouhidel K., personal communication) and in cells expressing other tonoplast proteins under the control of the 35S promoter [51]. The structures observed in *Arabidopsis *cotyledons [49] were called "bulbs", a term which reflects a spherical structure that is not entirely closed. The open section of these bulbs may be the result of incomplete vesicle formation [49]. The bulbs appear to be attached to the actin cytoskeleton, as actin inhibitor treatment allowed their immobilization and 3-D reconstructions [51]. The 3-D images of the spherical structures we observed in tobacco suspension cells cultured under dehydration stress are similar to the 3-D reconstructed cylindrical structures observed in GFP-AtVam3p expressing root protoplasts of *A. thaliana *[52]. Recently, fluorescent circular structures were seen in germinating pollen tubes of δ -TIP::GFP expressing *Arabidopsis *plants [20]. These mobile cytoplasmic invaginations may be a widespread characteristic of actively growing tissues.

Why do vacuoles of PEG acclimated suspension cells contain "bulbs"? PEG mimics dehydration when applied to the culture medium, resulting in a decrease of turgor. Similarly, cotyledons undergo a desiccation process. In *Spirodela intermedia *upper mesophyll cells, a breakdown of the tonoplast into small vesicles was observed after PEG inclusion [53]. Correlation of observed membrane fluorescence intensity with protein quantity suggests that the membrane of the spherical vacuolar structures is likely to contain twice the amount of aquaporins per surface area as the peripheral tonoplast, suggesting a higher level of water exchange. The spherical structures could reflect the presence of lipid domains in the tonoplast where aquaporins are concentrated and vesicle formation occurs. Indeed, some studies showed a high tonoplast fluidity [54], as well as a specific feature of fatty acid composition that may be responsible for the tonoplasts' unique fluidity and high elasticity [55] required for osmotic processes in the cell. Our results support the invagination model, postulated by Uemura and co-workers [52], where parts of the tonoplast form a double-layered membrane structure inside the vacuolar lumen. The spherical structures could serve as a reservoir, not only for membrane expansion, but also to allow for quicker homeostasis adjustments. A higher tonoplast surface area to cell volume ratio would greatly enhance the cells' capacity to maintain large ion pools during growth [56].

## Conclusion

The data presented in this study demonstrates the utility of the aquaporin BobTIP26-1::GFP as a powerful tool for visualizing 3-D membrane rearrangements within stressed tobacco cells. The technique used here provides highly resolved pictures and support of the notion that tobacco suspension cells contain a single major vacuole with a lytic function. Further investigations are required to establish the exact origin and function of the membrane enclosed, intra-vacuolar circular structures exhibited by tobacco cells under PEG mediated stress.

## Methods

### Plant material

Tobacco (*Nicotiana tabacum *L. var. Wisconsin 38) suspension cells expressing *BobTIP26-1::gfp *[17] were grown in MS medium [57], with regular shaking, at 24°C under constant photosynthetic illumination (200 μE·m^-2^·sec^-1^).

### Three-dimensional reconstruction

Three to seven day-old cells were analyzed under a Leica TCS 4D laser confocal microscope (Leica Microsystems, Wetzlar, Germany) equipped with an argon-krypton laser (488/515 BP-FITC). The laser was focused on individual cells through a 40x NA1 oil-immersion objective. For each cell, a stack of between 100 and 200 images was collected (resolution 256 × 256 with 0.50 to 0.65 μm of z-step). Merged individual confocal images (red and green channels) were composed using Corel Photo-Paint 7 software (Corel, Ottawa, Canada). To obtain 3-D reconstructions, confocal image stacks were imported into the three-dimensional visualization software Imaris 2.7 (Bitplane AG, Switzerland) running on a Silicon Graphics^® ^Octane2™ workstation (SGI, Paris, France). After baseline subtraction, a subregion was defined. Then, the isosurface module of Imaris was used to reconstitute the 3-D pictures. An adequate isovalue was defined for each channel prior to viewing of the computed surface using IvView. Isosurface rendered pictures were then stored as tiff files using the MediaRecorder media tool. Realistic on-screen representations of 3-D reconstructions were analyzed in depth with stereoscopic viewing devices (CrystalEyes^®^, StereoGraphics^®^).

### Cell perfusion

Cell perfusion was performed in a homemade perfusion chamber linked to a peristaltic pump (flux = 20 mL·h^-1^) (IBMP, Strasbourg, France). *BobTIP26-1::gfp *expressing cells were first adhered to poly-lysinized glass cover slips. The following solutions were used for cell perfusions: MS supplemented with either 0.17 M NaCl or 0.6 M mannitol or 0.5 M sorbitol. Plasmolysis and deplasmolysis were monitored using an inverted laser confocal microscope LSM510 (Zeiss Axiovert 100 M, Jena, Germany) fitted with a Zeiss 63X water-immersion objective. Confocal time lapse series were then collected. Osmolarities were measured with a Vapro^® ^vapor pressure osmometer (Model 5520; Wescor, Logan, UT, USA).

### Acclimation stress conditions

Five mL of *BobTIP26-1::gfp *expressing cells at exponential growth phase, were subcultured in MS medium supplemented with any of the following osmotica: 170 mM NaCl, 0.6 M mannitol, 0.5 M sorbitol or 10% PEG_8000_. The stressed and control (MS without an osmoticum) cells were analyzed over 7 days and 3-D reconstructed as described above. Fluorescent intensities were measured using IPLab software (Scanalytics, Fairfax, VA, USA).

## Authors' contributions

DR drafted the manuscript, carried out the experiments, including the microscopic observations and computer generated reconstructions. FM and NLC conceived of the study, and participated in its design and coordination. NLC also helped to draft the manuscript. All authors read and approved the final manuscript.

## Supplementary Material

Additional File 1 **Cellular tomogram through two overlapping *BobTIP26-1::gfp *expressing cells.** Click here for file

Additional File 2 **Animation of 3-D reconstructed vacuoles. **The reconstructed vacuoles from Figure 3d were rotated. Notice chloroplasts (red) on the outer sides of the vacuoles, within transvacuolar strands and the cells' peripheries. Click here for file

Additional File 3 **Cellular tomogram through a protoplast with a GFP-labeled tonoplast. **The protoplast was prepared from *BobTIP26-1::gfp *expressing cells as previously described [17]. Click here for file

Additional File 4 **Cellular tomogram through a plasmolysed cell demonstrating the intact tonoplast structure and its folding. **The tonoplast fluoresces in green. Hechtian strands are not labeled. Bar = 5 μm. Click here for file

Additional File 5 **Plasmolysis-deplasmolysis cycle of cells bathed with 0.5 M sorbitol. **Time is marked at the bottom left; changes of media are given at the top left (10 min: + 0.5 M sorbitol; 23.5 min: + normal MS medium). Bar = 20 μm. Click here for file
